# Peroxisome Proliferator Activated Receptors and Lipoprotein Metabolism

**DOI:** 10.1155/2008/132960

**Published:** 2007-11-21

**Authors:** Sander Kersten

**Affiliations:** Nutrigenomics Consortium and Nutrition, Metabolism and Genomics Group, Wageningen University, P.O. Box 8129, 6700 Wageningen, EV, The Netherlands

## Abstract

Plasma lipoproteins are responsible for carrying triglycerides and cholesterol in the blood and ensuring their delivery to target organs. Regulation of lipoprotein metabolism takes place at numerous levels including via changes in gene transcription. An important group of transcription factors that mediates the effect of dietary fatty acids and certain drugs on plasma lipoproteins are the peroxisome proliferator activated receptors (PPARs). Three PPAR isotypes can be distinguished, all of which have a major role in regulating lipoprotein metabolism. PPARα is the molecular target for the fibrate class of drugs. Activation of PPARα in mice and humans markedly reduces hepatic triglyceride production and promotes plasma triglyceride clearance, leading to a clinically significant reduction in plasma triglyceride levels. In addition, plasma high-density lipoprotein (HDL)-cholesterol levels are increased upon PPARα activation in humans. PPARγ is the molecular target for the thiazolidinedione class of drugs. Activation of PPARγ in mice and human is generally associated with a modest increase in plasma HDL-cholesterol and a decrease in plasma triglycerides. The latter effect is caused by an increase in lipoprotein lipase-dependent plasma triglyceride clearance. Analogous to PPARα, activation of PPARβ/δ leads to increased plasma HDL-cholesterol and decreased plasma triglyceride levels. In this paper, a fresh perspective on the relation between PPARs and lipoprotein metabolism is presented. The emphasis is on the physiological role of PPARs and the mechanisms underlying the effect of synthetic PPAR agonists on plasma lipoprotein levels.

## 1. INTRODUCTION

Plasma lipoproteins are responsible for carrying triglycerides and cholesterol in the blood and ensuring their delivery to target organs. Extensive research over the past few decades has demonstrated that elevated plasma levels of cholesterol-rich low-density lipoproteins (LDLs) are associated with increased risk for coronary heart disease, whereas elevated levels of high-density lipoproteins (HDLs) have a protective effect. Accordingly, raising HDL levels and especially lowering LDL levels has become the cornerstone for the nutritional and pharmacological prevention and treatment of coronary heart disease. While lowering of plasma LDL can be efficiently and adequately achieved by treatment with statins, limited pharmacological treatment options are available for efficiently raising HDL levels. Hence, the quest for effective and safe drugs that raise HDL levels and/or decrease the atherogenic properties of plasma lipoproteins continues. A group of proteins that plays a major role in the regulation of lipoprotein metabolism and can be considered as major drug targets for correcting abnormal plasma lipoprotein levels are the nuclear receptors [1]. Nuclear receptors are ligand-activated transcription factors that alter gene transcription by direct binding to specific DNA response elements in target genes [2]. In addition, they modulate transcription by interfering with specific intracellular signaling pathways, thereby impairing transcriptional activation by other transcription factors. Nuclear receptors share a common modular structure that includes a relatively well-conserved central DNA-binding domain and a C-terminal ligand binding domain (LBD) [2]. Several nuclear receptors have been shown to be involved in the regulation of plasma lipoprotein metabolism, including the estrogen receptors (ERs), the oxysterol receptors (LXRs), the bile acid receptor (FXR), and the fatty acid receptors (PPARs). Here, the emphasis will be on the role of PPARs.

The PPAR family includes three members encoded by distinct genes: α, β/δ, and γ [3]. Since the initial discovery of the PPARα isotype in 1990 [4], an impressive amount of literature on these receptors has accumulated. PPARs mainly operate by governing the expression of specific sets of genes. Analogous to many other nuclear receptors, PPARs bind to DNA and regulate transcription as a heterodimer with the retinoid X receptor (RXR) [5]. The genomic sequence recognized by PPARs, referred to as PPAR response element or PPRE, consists of a direct repeat of the consensus hexameric motif AGGTCA interspaced by a single nucleotide. Functional PPREs have been identified in genes involved in a variety of biological processes including lipid and glucose metabolism, detoxification, and inflammation [6]. Activation of transcription by PPARs is achieved by binding of specific ligands to the LBD, followed by recruitment of coactivator proteins and dissociation of corepressors. Coactivator recruitment generally leads to an increase in enzymatic activity of histone acetyltransferases, histone methyltransferases, and subsequent nucleosome remodeling, activities which are essential to initiate transcription of PPAR target genes. X-ray crystallographic analysis of the LBD of PPARs has revealed an exceptionally spacious ligand binding pocket that can be occupied by a wide variety of synthetic and natural agonists, including numerous fatty acids and fatty acid-derived eicosanoids [7, 8].

The three PPARs are distinguishable by specific tissue and developmental patterns of expression and by their activation by distinct, yet overlapping, ligands [9]. The PPARα isotype is well expressed in tissues such as liver, heart, and small intestine and regulates a variety of target genes involved in cellular lipid metabolism ranging from mitochondrial, peroxisomal, and microsomal fatty acid oxidation to fatty acid uptake and binding, lipolysis, lipogenesis, and glycerol metabolism [6]. In contrast, PPARγ, which is highly expressed in brown and white adipose tissue, directs the expression of genes involved in adipocyte differentiation and fat storage. In addition, PPARγ governs glucose uptake and storage [10]. Much less is known about the ubiquitously expressed PPARβ/δ, although recent evidence suggests an involvement in wound healing [11], fatty acid oxidation [12], and lipoprotein metabolism [13].

Here we present an overview of the literature on PPARs and lipoprotein metabolism. The emphasis is on physiological role of PPARs and the mechanisms underlying the effect of synthetic PPAR agonists on plasma lipoproteins.

## 2. PPARα AND PLASMA TRIGLYCERIDE METABOLISM

The seminal evidence that placed PPARα at the center of lipoprotein metabolism was the demonstration that fibrates, which had been used clinically for many years to treat dyslipidemia, act by binding to PPARα and induce PPARα-dependent gene transcription [4, 14]. The role of PPARα in lipoprotein metabolism could thus be extrapolated retrospectively by analyzing the reported effect of fibrates. The availability of PPARα null mice further spurred progress in elucidating PPARα function and has resulted in an extensive picture of the role of PPARα in lipoprotein metabolism [15].

Numerous clinical studies in humans have provided ample evidence that fibrates, which include clofibrate, bezafibrate, fenofibrate, and gemfibrozil, effectively lower fasting plasma triglycerides (TG) [16–19]. The plasma TG lowering effect of fibrates can be reproduced in mice [20, 21]. Conversely, plasma TG levels are elevated in mice lacking PPARα [22]. Since in the fasted state plasma TG are carried mainly in the form of very low-density lipoproteins (VLDL), this suggests that PPARα suppresses VLDL production in liver and/or stimulates clearance of VLDL triglycerides in peripheral tissues.

## 3. PPARα AND VLDL PRODUCTION

**Figure 1:**
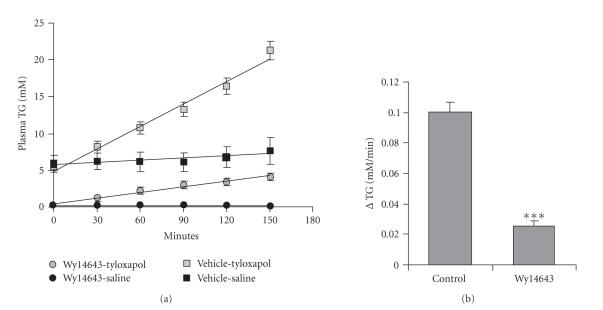
The PPARα agonist Wy14643 dramatically lowers VLDL production in a mouse model of hypertriglyceridemia. Male Angptl4-transgenic mice (n=7 per group) were given vehicle or Wy14643 for 10 days (0.1% mixed in their food). After a 24-hour fast, the LPL-inhibitor tyloxapol (Triton WR1339, 500 mg/kg bodyweight as 15% solution in saline) or saline were injected intraorbitally. (a) Plasma triglyceride concentration was measured every 30 minutes to determine the VLDL production rate. (b) Mean rate of increase of plasma TG concentration in mice that received tyloxapol. Differences were evaluated by Student’s t-test (***P<.001). Error bars represent SEM.

Limited data are available on the effect of fibrates on production and secretion of VLDL in humans. In one study, the PPARα agonist gemfibrozil decreased production of VLDL-TG, while clofibrate had no effect [23]. In mice, PPARα has been shown to have a major impact on hepatic TG secretion. Indeed, deletion of PPARα is associated with a significant increase in VLDL-TG production in liver [24, 25]. In contrast, activation of PPARα using Wy14643 dramatically lowers VLDL-TG production (Figure 1). Furthermore, activation of PPARα suppresses TG secretion from primary rat hepatocytes [26].

VLDL is synthesized by the stepwise lipidation of the structural component apolipoprotein B through the action of microsomal triglyceride transfer protein (MTTP), resulting in the gradual formation of a mature TG-rich VLDL1 particle [27]. It may be expected that elevated hepatic TG levels increase VLDL secretion, on the one hand by targeting apolipoprotein B away from degradation toward secretion, thus increasing VLDL particle number, and on the other hand by increasing the amount of TG that becomes incorporated into VLDL, thus increasing VLDL particle size [27–29]. However, a positive correlation between hepatic TG and VLDL production is not always evident, as illustrated by the lack of change in hepatic VLDL production in ob/ob mice despite massive steatosis [30]. These data feed a growing recognition that the relation between hepatic TG storage and VLDL production is dependent on where the excess TG are stored. This argument holds both at the tissue level, as only excess TG stored in the periportal area may promote VLDL formation, and at the cellular level, as TG incorporated into VLDL are likely drawn from specific intracellular lipid compartments.

**Figure 2:**
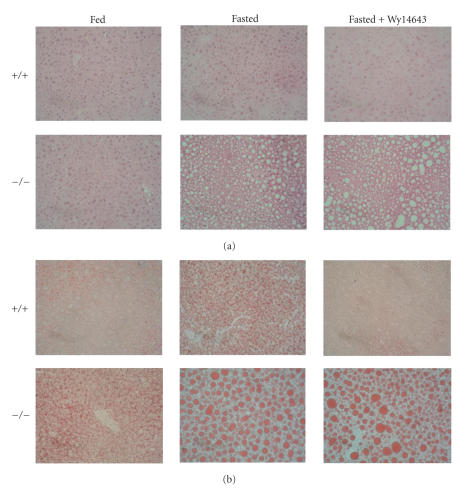
The PPARα agonist Wy14643 prevents the fasting-induced increase in liver TG levels. Male wild-type and PPARα null mice (n=5 per group) were given vehicle or Wy14643 for 10 days (0.1% mixed in their food). After a 24-hour fast, livers were dissected and stained histochemically using hematoxylin/eosin (a) or oil Red O (b). Representative livers sections are shown. Differences visualized by histochemistry were perfectly confirmed by quantitative measurement of hepatic TG levels.

Numerous studies have shown that PPARα activation lowers liver TG levels, especially in the context of a fatty liver [31–36]. Conversely, deletion of PPARα is associated with elevated hepatic TG stores, which is evident under normal fed conditions but becomes considerably more pronounced after prolonged fasting and chronic high fat feeding [22, 37–40]. The potent effect of PPARα activation and deletion on hepatic TG levels is illustrated in Figure 2. Remarkably, treatment of wildtype but not PPARα null mice with Wy14643 for 10 days can completely prevent the fasting-induced increase in hepatic TG, most likely by stimulating fatty acid oxidation. Indeed, probably the best understood property of PPARα is its ability to stimulate fatty acid oxidation by upregulating almost every single gene within the mitochondrial, peroxisomal, and microsomal fatty acid oxidation pathway, including carnitine palmitoyl transferase 1 and 2, acyl-CoA oxidase, acyl-CoA dehydrogenases, and numerous others [6]. Many of these genes have been identified as direct PPARα targets characterized by the presence of a functional PPRE. Accordingly, the most plausible explanation for the hepatic TG lowering effect of PPARα activation is that by promoting fatty acid oxidation, PPARα shifts fatty acids away from esterification and storage. While its effect on fatty acid oxidation likely accounts for the major share of its antisteatotic action, regulation of other genes and pathways by PPARα may contribute to some extent as well.

Suppression of VLDL production by PPARα agonists is generally attributed to lowering of hepatic TG stores, despite uncertainties surrounding the relationship between hepatic TG storage and VLDL production. In addition to its role in fatty acid catabolism, PPARα impacts on multiple aspects of intracellular lipid trafficking and metabolism, some of which may oppose hepatic TG lowering, including induction of genes involved in fatty acid synthesis and fatty acid elongation/desaturation [41–44]. Furthermore, expression of MTTP, which is involved in the lipidation of apoB100 to form a nascent VLDL particle, has recently been shown to be increased by PPARα [21]. Upregulation of MTTP may promote apoB100 secretion, which together with a decreased TG availability may favor the targeting of apoB100 to IDL and LDL rather than VLDL [21]. Interestingly, a recent study points to adipose differentiation-related protein (ADRP), which is a direct target gene of PPARα [45], as a potential mediator of the effect of PPARα on VLDL production. Using cultured cells it was shown that an increase in ADRP prevents the formation of VLDL by diverting fatty acids from the VLDL assembly pathway into cytosolic lipid droplets [46]. It can be expected that as the process of VLDL assembly and secretion becomes better understood and the role of PPARα in this process is further clarified, the general view on the mechanism underlying the effect of PPARα on hepatic VLDL secretion may change.

## 4. PPARα AND VLDL-TG CLEARANCE

Several studies have examined the impact of PPARα on clearance of TG-rich lipoproteins in humans, all of which show increased clearance after treatment with PPARα agonists [23, 47–49]. Clearance of VLDL-TG from plasma is mediated by the enzyme lipoprotein lipase (LPL) which thus has a critical role in determining plasma TG concentrations. LPL is synthesized mainly by adipocytes and myocytes, and after translocation to capillary endothelial cells it is anchored into the vessel wall via heparin-sulphate proteoglycans. Treatment of human subjects with PPARα agonists is associated with a significant increase in postheparin total LPL activity, suggesting that stimulation of plasma TG clearance by PPARα agonists can be attributed to enhanced LPL activity [49–51].

Theoretically, changes in LPL activity can be achieved by altering the production of LPL itself, or by altering the production of proteins that assist with LPL function or modulate its enzymatic activity. The latter group includes apolipoproteins such as APOC3, APOC2, and APOA5, as well as angiopoietin-like proteins 3 and 4. While it is clear that expression of LPL is upregulated by PPARα in liver [52], no evidence is available indicating a role for PPARα in governing LPL expression in heart and skeletal muscle. According to our unpublished microarray data, neither PPARα deletion nor 5-day treatment with Wy14643 had any influence on LPL mRNA expression in mouse heart. It thus appears that rather than by regulating expression of LPL itself, PPARα agonists stimulate plasma TG clearance by altering the hepatic expression of inhibitors or activators of LPL activity. In both mouse and human, hepatic mRNA expression and plasma levels of APOC3, which inhibits LPL activity, are lowered by PPARα agonists [53–56]. Several mechanism have been put forward to explain downregulation of APOC3 expression by PPARα, involving the transcription factors Rev-erbα, HNF4α, or FOXO1 [57–60]. In contrast to APOC3, PPARα agonists increase hepatic expression and plasma levels of APOA5, an activator of LPL [61]. A functional PPAR responsive element has been identified in the promoter of the human APOA5 gene, classifying APOA5 as a direct PPARα target gene [62, 63].

It can be hypothesized that the stimulatory effect of PPARα on clearance of TG-rich lipoproteins may be counterbalanced by PPARα-dependent upregulation of the LPL inhibitor Angptl4 [64, 65]. Plasma levels of Angptl4 are increased by fenofibrate treatment [66]. Data obtained from various transgenic mouse models and from human genetic studies indicate that Angptl4 inhibits the clearance of TG-rich lipoproteins, likely by stimulating the conversion of catalytically active dimeric LPL to catalytically inactive monomeric LPL [67–72]. It can be speculated that upregulation of Angptl4 may explain the inhibitory effect of PPARα agonists on LPL activity in macrophages, adipose tissue, and cardiomyocytes [73–76].

## 5. PPARα AND HDL METABOLISM

In addition to their plasma TG-lowering effect, fibrates are used clinically for their ability to raise plasma HDL-cholesterol (HDLc) levels. A recent meta-analysis of 53 clinical trials indicates that on average, fibrates elevate plasma HDLc levels by about 10%, which translates into a 25% reduction in risk for major coronary events [77]. Remarkably, this effect is not observed in rodents, which seriously complicates study of the molecular mechanisms underlying the effect of PPARα agonists on HDL. In mice, plasma total cholesterol and HDLc levels are reduced by treatment with synthetic PPARα agonists [78], whereas levels are increased in mice lacking PPARα [20]. The differential effects of PPARα on plasma HDL between mouse and human is likely due to species-specific regulation of apolipoprotein A-I (APOA1), the core apolipoprotein of HDL. Whereas PPARα activation increases plasma levels and hepatic mRNA expression of APOA1 in human, as supported by studies using human APOA1 transgenic mice and human hepatocytes [79], the opposite effect is observed in rodents [78]. The lack of upregulation of APOA1 gene expression by PPARα in rat was attributed to 3 nucleotide differences between the rat and the human APOA1 promoter A site, rendering a positive PPAR-response element in the human APOA1 promoter nonfunctional in rats [80]. In addition to APOA1, plasma levels and hepatic mRNA expression of APOA2, another major apolipoprotein component of HDL, are also increased by fibrates in humans [51, 81]. In contrast, in rodents both a reduction and induction of APOA2 expression after treatment with fibrates have been observed [20, 78].

In recent years, our knowledge regarding the mechanisms and location of HDL formation has improved considerably. Recent evidence suggests that the intestine and liver are responsible for the major share of HDL synthesis [82, 83]. It is generally believed that HDL is formed by lipidation of lipid poor APOA1 mediated by the cholesterol efflux transporter ABCA1. The importance of ABCA1 in HDL synthesis is illustrated by the almost complete absence of HDL from plasma of patients with a dysfunctional ABCA1 gene [84]. This metabolic abnormality is reproduced in mice that lack ABCA1 [85, 86]. Importantly, the expression of ABCA1 is increased by PPARα in intestine and macrophages [87, 88]. No PPRE has yet been identified in the human or mouse ABCA1 gene, suggesting that ABCA1 may not be a direct PPARα target. Instead, upregulation of ABCA1 mRNA by PPARα agonists in macrophages likely occurs via PPARα-dependent upregulation of LXR, which is a transcriptional activator of ABCA1 [88, 89]. Whether the same mechanism operates in intestine remains unclear.

Recently, ABCG1 was identified as the transporter responsible for cellular efflux of cholesterol towards mature HDL [90]. So far no evidence is available that suggests regulation of ABCG1 by PPARs.

Several proteins are involved in HDL remodeling including lecithin cholesterol acyltransferase (LCAT), phospholipid transfer protein (PLTP), and cholesteryl ester transfer protein (CETP). In mice, fibrates decrease plasma LCAT activity and hepatic LCAT mRNA expression [91]. Hepatic expression and plasma activity of PLTP, which increases HDL particle size by catalyzing the transfer of phospholipids from VLDL/IDL to HDL, are increased by PPARα in wild-type but not PPARα null mice. Accordingly, upregulation of PLTP may account for the observed increase in HDL particle size in mice treated with fibrates [92]. Since CETP is absent from mice, the role of PPARα in the regulation of CETP activity has largely remained elusive. Interestingly, in a recent study using hCETP-transgenic mice on an apoE3 Leiden background, it was found that fenofibrate markedly reduced CETP activity in parallel with an increase in plasma HDLc levels [93]. These data imply that fibrates reduce CETP activity in humans, suggesting that the effect of fibrates on plasma HDL levels in humans may be partially achieved by suppressing CETP activity. In addition, it can be speculated that as PPARα activation decreases plasma VLDL levels, the acceptor pool for the CETP-catalyzed exchange of cholesterol-esters with HDL will be diminished, resulting in increased HDL size.

HDL cholesterol can also be cleared by the SCARB1-mediated selective removal of cholesterol from the HDL particle, or by endocytic uptake and degradation of the whole particle, called holoparticle HDL uptake. A possible mechanism by which fibrates may impair HDL clearance is by downregulating hepatic SCARB1 gene expression in a PPARα dependent manner [94].

## 6. PPARβ/δ AND LIPOPROTEIN METABOLISM

While the role of PPARα in the regulation of lipoprotein metabolism is relatively well characterized, much less is known about PPARβ/δ. Initial studies in mice showed that selective PPARβ/δ agonists raise plasma HDLc levels [13, 95]. The HDLc-raising effect is also evident in rhesus monkeys [96], and, according to a recent report, in human subjects [97]. In monkey and human, but seemingly not in mice, PPARβ/δ agonists decrease plasma TG levels as well. The mechanism behind the HDLc-raising effect of PPARβ/δ agonists remains obscure, although a role for ABCA1, which is upregulated by PPARβ/δ, has been proposed [96].

In line with the plasma TG-lowering effect of PPARβ/δ agonists observed in primates, plasma TG levels are elevated in PPARβ/δ null mice [98]. In contrast, plasma total cholesterol and HDLc remain unchanged. It was proposed that the elevated plasma TG levels in PPARβ/δ null mice are caused by a combination of increased VLDL production and decreased plasma TG clearance, as evidenced by a decrease in postheparin LPL activity and increased hepatic expression of LPL inhibitors Angptl3 and 4. Overall, insight into the molecular mechanisms that may underlie the observed changes in plasma lipoproteins is lacking, which is partly due to the fact that very few PPARβ/δ specific or selective target genes are known. Since PPARα agonists also increase plasma HDLc levels, it might be hypothesized that PPARβ/δ agonists might act via common molecular targets. However, at least in mice, PPARα and PPARβ/δ agonists display divergent effect on plasma TG levels, suggesting a different mode of action.

## 7. PPARγ AND PLASMA TRIGLYCERIDE METABOLISM

Synthetic PPARγ agonists are prescribed for their ability to promote insulin sensitivity and lower plasma glucose levels in patients suffering from type 2 diabetes mellitus (T2DM). On top of an insulin-sensitizing action, numerous studies in mice and humans have shown that use of PPARγ agonists leads to a reduction in fasting and postprandial plasma TG levels [99–103]. Some variability in the plasma TG lowering effect is observed between different PPARγ agonists, and in mice between various disease models. Indeed, no effect of PPARγ agonists on plasma TG is observed in the two mouse models most commonly used for atherosclerosis research, which are the LDL receptor knock-out and apoE knock-out mice [104]. In humans rosiglitazone seems to specifically lower postprandial but not fasting TG levels [105, 106]. Evidence has been provided that the plasma TG lowering effect of PPARγ agonists may be connected to their insulin sensitizing action by suppressing adipose tissue lipolysis and plasma FFA levels, which is expected to lead to decreased hepatic VLDL-TG production [106]. However, no relationship exists between the insulin-sensitizing potency of PPARγ agonists and plasma TG lowering [107]. Furthermore, in a recent study, treatment of type 2 diabetic subjects with pioglitazone did not result in any change in hepatic VLDL-TG production [108]. Thus, whereas PPARα agonist lowers plasma TG by a combination of suppressing hepatic VLDL production and stimulating plasma TG clearance, PPARγ agonists seem to lower plasma TG exclusively by enhancing plasma TG clearance [100, 108].

The stimulatory effect of PPARγ agonists on plasma TG clearance is achieved by upregulating LPL expression and activity in adipose tissue [52, 100, 106, 109, 110], which is associated with an increase in postheparin plasma LPL mass/total activity [101, 102]. As a consequence, LPL-mediated lipolysis and the fractional clearance rate of VLDL-TG are elevated [108]. Besides directly regulating LPL production, PPARγ agonists may influence LPL-mediated lipolysis by decreasing plasma levels of APOC3, a potent inhibitor of LPL [108].

Interestingly, in rats induction of LPL activity and gene expression by PPARγ agonist was observed in inguinal but not retroperitoneal adipose tissue [111]. This type of adipose depot-specific regulation of LPL by PPARγ likely accounts for the redistribution of stored fat from visceral towards subcutaneous adipose tissue upon treatment with PPARγ agonists [112]. Also, no induction of LPL expression by PPARγ was observed in murine skeletal muscle [113].

In contrast to what is observed in vivo, PPARγ agonists decrease LPL activity in primary rat and mouse 3T3 adipocytes [100, 114]. It can be hypothesized that the inhibition of LPL activity may be mediated by upregulation of Angptl4, similar to what was discussed for the suppression of LPL activity in various cell types after treatment with PPARα agonist. In light of the recent finding that rosiglitazone raises plasma Angptl4 levels in human subjects [115], and that Angptl4 increases abundance of monomeric LPL in preheparin plasma (our unpublished data), it can be speculated that upregulation of Angptl4 may also account for the observed increase in plasma preheparin LPL levels in subjects treated with pioglitazone [108].

Use of gene targeting to study of the role of PPARγ in regulation of lipoprotein metabolism has been complicated by the lethality of PPARγ null mice. However, mice with a specific ablation of the PPARγ2 isoform are viable and, opposite to the effect of PPARγ agonists, show elevated plasma TG levels, especially on a leptin-deficient background [116]. A similar elevation of plasma TG was observed in mice in which PPARγ was specifically deleted in adipose tissue [117].

Apart from LPL, very few PPARγ target genes that impact on TG-rich lipoproteins are known. It has been shown that LDL-receptor-related protein 1 (LRP-1), which is involved in clearance of cholesteryl-esters from chylomicron remnants and possibly HDL, is a direct target gene of PPARγ in human adipocytes [118]. These data suggest that upregulation of LRP-1 may contribute to the stimulatory effect of PPARγ agonists on clearance of TG-rich lipoproteins.

## 8. PPARγ AND HDL METABOLISM

Although PPARγ agonists are best known for their ability to lower plasma glucose and TG levels, depending on the type of PPARγ agonist and the type of animal species/model, plasma levels of cholesterol and specific lipoprotein subclasses may be altered as well [104, 119]. Recently, the results of two large clinical trials involving either rosiglitazone or pioglitazone were reported. In the ADOPT trial, 4360 subjects recently diagnosed with T2DM were randomly assigned to treatment with metformin, glyburide, or rosiglitazone. After 4 years, plasma HDLc levels were modestly higher in the rosiglitazone-treated patients [120]. In the proactive trial, 5238 patients with type 2 diabetes received either pioglitazone or placebo. Again, a significant increase in plasma HDLc levels was observed in the patients treated with pioglitazone [121]. The small but reproducible increase in plasma HDLc upon treatment with PPARγ agonists was substantiated in a meta-analysis summarizing the effects of thiazolidinediones from a large number of randomized controlled trails [122]. In addition, treatment with PPARγ agonists is associated with an increase in LDL size [101, 103, 119]. It has been reported that the relative efficacy of pioglitazone towards ameliorating plasma lipid levels is more favorable compared to rosiglitazone [119].

Presently, the mechanism(s) behind the HDLc raising effect of PPARγ agonists remains elusive. Possibly, PPARγ agonists may carry minor agonist activity towards PPARα. However, in contrast to PPARα agonists, PPARγ agonists do not appear to have any effect of APOA1 and APOA2 syntheses [100, 108]. The observation that plasma HDLc levels do not respond to PPARγ agonist treatment in rodents complicates study of the underlying mechanisms [100]. It is conceivable that the modest increase in HDLc following PPARγ agonist treatment is due to reduced CETP-mediated exchange of VLDL TGs for HDL cholesterol, concomitant with a drop in VLDL-TG levels. Finally, PPARγ has been shown to upregulate expression of ABCA1 in macrophages [88, 123]. As ABCA1 is required for the flux of cholesterol from cells onto APOA1 to form nascent HDL, upregulation of ABCA1 by PPARγ may contribute to the HDLc-raising effect of PPARγ. However, it still needs to be demonstrated that expression of ABCA1 is under control of PPARγ in tissues responsible for the major share of HDL synthesis, which are intestine and liver.

## 9. CONCLUSION

PPARs have a major impact on levels of lipoproteins in plasma by governing the expression of numerous genes involved in the synthesis, remodeling, and clearance of plasma lipids and lipoproteins. The changes in plasma lipoprotein levels associated with treatment with PPAR agonists, characterized by decreased plasma TG levels, increased HDLc, and an increase in LDL size, are expected to decrease the risk for cardiovascular disease. In recent years, several new proteins that play a role in lipoprotein metabolism have been identified. In addition, the functional characterization of other proteins involved in lipoprotein metabolism has advanced significantly. As progress is made in PPAR-dependent gene regulation, especially in human, our insight into the molecular mechanisms underlying the effects of PPARs on plasma lipoproteins will further continue to improve.
